# ‘You don't know which bits to believe’: qualitative study exploring carers’ experiences of seeking information on the internet about childhood eczema

**DOI:** 10.1136/bmjopen-2014-006339

**Published:** 2015-04-08

**Authors:** Miriam Santer, Ingrid Muller, Lucy Yardley, Hana Burgess, Steven J Ersser, Sue Lewis-Jones, Paul Little

**Affiliations:** 1 Department of Primary Care and Population Sciences, University of Southampton, Southampton, UK; 2 Faculty of Social and Human Sciences, University of Southampton, Southampton, UK; 3 School of Healthcare, University of Leeds, Leeds, UK; 4 Department of Dermatology, Ninewells Hospital & Medical School, Dundee, UK

**Keywords:** QUALITATIVE RESEARCH, PRIMARY CARE, PAEDIATRICS

## Abstract

**Objective:**

We sought to explore parents and carers’ experiences of searching for information about childhood eczema on the internet.

**Design:**

A qualitative interview study was carried out among carers of children aged 5 years or less with a recorded diagnosis of eczema. The main focus of the study was to explore carers’ beliefs and understandings around eczema and its treatment. As part of this, we explored experiences of formal and informal information seeking about childhood eczema. Transcripts of interviews were analysed thematically.

**Setting:**

Participants were recruited from six general practices in South West England.

**Participants:**

Interviews were carried out with 31 parents from 28 families.

**Results:**

Experiences of searching for eczema information on the internet varied widely. A few interviewees were able to navigate through the internet and find the specific information they were looking for (for instance about treatments their child had been prescribed), but more found searching for eczema information online to be a bewildering experience. Some could find no information of relevance to them, whereas others found the volume of different information sources overwhelming. Some said that they were unsure how to evaluate online information or that they were wary of commercial interests behind some information sources. Interviewees said that they would welcome more signposting towards high quality information from their healthcare providers.

**Conclusions:**

We found very mixed experiences of seeking eczema information on the internet; but many participants in this study found this to be frustrating and confusing. Healthcare professionals and healthcare systems have a role to play in helping people with long-term health conditions and their carers find reliable online information to support them with self-care.

**Strengths and limitations of this study**

- This article explores the experiences of a relatively educated group of parents and carers, and found that some encountered significant barriers to finding useful online healthcare information about a common long-term condition.
- Experiences varied widely, with some finding too little, others finding too much and others finding information, but not knowing whether it was trustworthy.
- Most people who had sought online information encountered difficulties and would have welcomed more signposting from healthcare professionals.
- A limitation of this study is that several participants had not sought information online for their child's eczema, either because they had obtained sufficient information elsewhere or because their child's eczema was mild.
- A more diverse sample of interviewees might have uncovered a still wider range of information-seeking experiences.

## Background

Medical information available on the internet is a great resource for patients and carers, and access to this information continues to improve. The quantity of health–related information continues to increase, but concerns remain about: the quality of information available;^1^ consumers’ ability to make sense of this information;^2^ and the potential threat to the doctor–patient relationship from web-derived information.^3^

Quality criteria for health information have been available for several years,^4^ yet studies that have systematically reviewed the quality of online information have found this to be variable.^5^ ^6^ Consumers may be aware of how to check for the credibility of online data sources, but it seems that they do not always do so and tend not to recall the provenance of particular pieces of information.^2^ Health literacy is an important factor in how successful people are at using online information as this affects their ability to access, understand and appraise health information.^7^ Doctors can feel challenged by patients bringing web-derived information to the consultation,^8^ ^9^ although it seems that this is not necessarily problematic and techniques to deal with such challenges can be learned. Patients and carers view the search for web-based information as part of taking responsibility for their own health and many value the opportunity to discuss this with healthcare providers.^9^ ^10^ Although accessing online information may help people making health-related decisions, the physician generally remains the primary source of information and advice.^11^

Most patients seek health-related information online at some point.^12^ Parents of young children are often particularly active seekers of web-based health information,^13^ yet relatively little is known about their experiences of this. Parents of young children with eczema may be especially likely to seek information as eczema can cause significant distress to the child and family, for instance due to sleep disturbance and itch.^14^ ^15^

We carried out a qualitative study among parents and carers of children with eczema, the primary aim of which was to explore their understandings of eczema and its treatment. As part of this we asked participants where they had sought and obtained information from. We present our findings regarding carers’ experiences of searching for information about childhood eczema on the internet.

## Methods

The methods have been described in detail elsewhere^16^ and are summarised here. Interviewees were recruited from six general practices in the South of England. Primary care staff searched their databases for children aged 5 years or less with a recorded diagnosis of eczema (Read codes: atopic eczema/dermatitis, infantile eczema or eczema not otherwise specified) and posted invitations to participate to ‘parent or carer of (name of child)’. Carers were asked to return a reply slip to the research team and if they indicated that they would like to participate and that their child's eczema was still a problem, they were telephoned to arrange an interview. We attempted to obtain views of participants from a range of socioeconomic backgrounds by sampling from practices with varying deprivation scores.

Qualitative interviews were semistructured and followed an interview guide developed from the literature and study rationale (box 1). Interviews lasted between 30 and 60 min and were carried out in participants’ homes, except for one, where the participant preferred to be interviewed at her health centre. Interviews were carried out by MS, CH and HB, from December 2010 to May 2011. Interviewers were not previously known to participants and introduced themselves as ‘researchers’, except for CH who introduced herself as a medical student. MS and HB were working as academic GPs, but did not inform patients that they were GPs at the start of the study in case this influenced their responses; the research was not carried out in practices where they worked. If participants asked questions about eczema management then they were asked to save these until the end of the interview. In some cases it arose in the discussion after the interview that the interviewer was a general practitioner (GP) as well as a researcher. Interviews were audiorecorded, transcribed and pseudonymised. Data saturation was achieved for themes regarding beliefs and understandings around childhood eczema and eczema treatment, although saturation may not have been achieved for themes around seeking information online.

Box 1 **Interview guide**

Can you tell me a bit about your child's eczema to start with?

- How does it affect your child?
- How does it affect family life?
- Can you tell me about what it was like when it first started?
- Can you tell me about how it has been more recently?

Can you tell me about any professional advice you have had about it?

Can you tell me about any other kinds of advice or information you have had? (other people? books, leaflets, internet?)

What kinds of things do you do to try to cope with the eczema?

- How do you feel about that?
- Can you tell me about any problems you have had with that?

Do you do anything else to try and help your child's eczema?

- Can you tell me about any difficulties you have doing these things?
- If so, how do you get round these difficulties?

How do other members of the family feel about the eczema?

What else do you think would be helpful or would have been helpful in the past?

Analysis was carried out thematically and iteratively using content analysis and a constant comparative approach.^17^ The software QSR NVivo V.9 facilitated the coding and organisation of data. A coding frame was developed based on codes arising from the first 10 transcripts by MS, HB and LY. MS and HB coded all transcripts, and further developed codes based on subsequent transcripts and further discussions with all authors. Data retrievals were carried out on the basis of codes, summaries written for each and tabulations made of interviewee characteristics in relation to these.

## Results

Invitations were sent to 289 households. We received 70 replies, of which 33 said eczema was no longer a problem, 3 declined for other reasons and 6 were not contactable, resulting in 28 interviews (10% of mail-out). Participant characteristics are shown in table 1. All participants were parents so we will present our findings with reference to ‘parents’ rather than ‘carers’.

**Table 1:** Participant characteristics

Participant characteristic	Description		
Interviewee's relationship to child*	27 mothers	4 fathers	
Interviewee's age	Median 36 years old (range 26 years to 46 years)		
Age of most affected child	Median 3 years (range 7 months old to 5 years old)		
Eczema severity (parent asked if mild, moderate or severe)	16 mild	10 moderate	2 severe
Consulting history	14 GP only	5 Dermatology nurse	9 Dermatology and/or allergy clinic
Family structure	Number of children in household median 2 (range 1 to 4); 27 households with both parents, 1 household with mother only		
Interviewees’ employment status*	14 full-time carers; 8 professional; 8 other such as admin or retail; 1 student		
Ethnicity*	25 white British; 3 black or Asian British; 2 white non-British; one mixed heritage British		

*Total 31 interviewees from 28 families as three couples were interviewed together. GP, general practitioner.

Interviewees generally expressed positive views about using the internet to seek health-related information and 14 of the 28 families interviewed said that they had sought information about eczema on the web. Several interviewees said that they would have looked for information had the condition been more severe.

I didn’t feel the need to source any more information about children’s eczema, ‘cos I know they grow out of it sometimes, ‘cos it wasn’t so severe. It didn’t have a huge impact on us, so there wasn’t the need to gain all the information that, you know, I probably would have needed if it was quite a bad case. (Julie)

Other interviewees said they had not sought information because they had a strong family history of eczema and therefore, were familiar with treatments or had access to information through their network of family and friends for some other reason.

### Experience of seeking information about eczema on the internet

#### ‘There wasn't really a lot about it’

Of the 14 families who had used the internet, most found this frustrating in several instances because they could not find any information that seemed relevant to them. For instance, Paul and Ali, who were interviewed together about their daughter's eczema, had been unsuccessful in finding any information that was useful to them on the internet.

Paul: We were looking for a bit of advice like on the internet to see what was on there to sort of calm her skin down a bit, because, if I remember rightly, it was when we were up for 2 or 3 nights because every time she laid down she would wake up because everything was sore and the doctor he kept changing the creams back and forth, back and forth.

Ali: It just seemed like nothing was helping.

Paul: And no-one was helping and we just got to the point where—oh, the internet, we'll just try that, but even on there, there wasn't really a lot about it, was there?

#### ‘There's so much… you don't know which bits to believe’

Some parents had found a lack of relevant information on the internet while others, conversely, found the volume of information so difficult to navigate that it was not useful to them or that they had ‘got lost’ among the volume of information. The effect of this was that they ‘gave up’ looking online and sought information elsewhere.

The trouble is, you go and gather so much data from other stuff, newspapers, websites, and all the rest of it, but you don't know which bits to believe. (Kim)

Actually, originally, before going to the GP, I did look on the internet for eczema; there's like so many types apparently, and so many … yeah there were, I think, too many types and now knowing exactly what was the one that affected him, I sort of got a bit lost really and thought, well why am I going to be reading about blah, blah, blah, eczema … might kill you or something and start worrying about that, when it might not be what he's got. So … that's when I thought I'll wait for the GP and see what he says. (Sophie)

#### ‘It depends who pays for whatever’

Some interviewees mentioned that they had come across expensive products for sale for eczema, for instance clothing, creams or online allergy tests, and that this was a further source of uncertainty. Some were aware of marketing influencing online information and questioned the credibility of sources. Despite this, there was no mention of differentiating between sources in terms of reliability of claims or checking the provenance of online information.

People, if they've got a lot of money, or people might not have that much money, they could be spending like £20, £30 on a pot of cream and it will do nothing. (Carrie)

I guess like everything with Google, it depends who pays for whatever will appear first and you always get lost in the thousand and something websites that appear. (Sophie)

Several people had come across products for sale on the internet for which there is no evidence base and they differed in how they reacted to this. One father had bought an Aloe Vera cream online, for instance, and was quite happy with this. Others felt less confident making purchases online, unsure whether to believe their claims for success or unsure whether they were legitimate treatments.

I’ve researched some things, some creams and stuff like—there was this nurse that created this cream—we didn’t get it yet, but I was thinking about getting a sample of it to try… but you don’t know. You could get a cream from a website and it could work wonders, or it might not. It could be fake, couldn’t it, you don’t know because they’re not licensed are they, some of these things. (Carrie)

Many parents were interested in allergy testing as a potential cure for their child's eczema.^18^ Parents had received conflicting messages about this and did not find that information on the internet clarified this.

A lot of people have mentioned allergy testing. I haven’t really looked into it because I'm a bit—sort of—there are so many of them; you go online and you type in allergy testing and then you are just bombarded with them. So what do I do, which one do I pick? There's so much to read and so little time, because obviously you’ve got children, you can't sit there reading paragraphs and paragraphs of information. (Fazeela)

#### ‘The internet was fantastic’

Some parents seemed quite confident in their use of the internet and said that they felt able to assess information and whether it was useful to them or not. Some said that they had looked for specific pieces of advice, for instance about clothing or laundry, whereas others had checked for further information about prescribed creams that they wanted more information about.

Until we had him, I didn't know very much about eczema at all, so it's good to know these things and to be able to access information easily. The Internet was fantastic for that, you can look at anything you like and then you just either take it on board or disregard it, depending on what you’re looking at. (Julie)

#### ‘Knowing that you're not on your own’

Some parents were not looking for information on the web, but found the online presence of other parents in a similar situation to be helpful; for instance, for reassuring them that others were going through similar difficulties or viewing the internet as a source of potential support.

I think what's useful for me is other people's experiences and just knowing that you're not on your own, when you're kind of thinking oh, I can’t get on top of it, what other people have done and tried, so that you can try it… (Caroline)

### Health professionals and the internet

The possibility of healthcare practitioners helping to direct people towards useful sources of online information was mentioned in some interviews, even though we did not specifically ask interviewees about this. Those who did mention it said that they would have welcomed direction towards online resources from health professionals.

But, I don't know, like the information I got from the GP at the time that she printed it… it was basic…. That doctor that gave me that printout could have given me that printout and said, look there's also more information on this website and maybe then—being the ones to tell everyone, really, because if you just expect people like me that hasn't got a clue do a search and, just get lost and never find anything. (Sophie)

If I just googled eczema or something like that, that I could look at it, but if I… went to see my GP or—I don't know, another health—or a practice nurse or something, that they also would say … this is a really good central kind of … all the up to date advice is all in this one place for you to go and look at. (Nicky)

## Discussion

People had very mixed experiences of using the internet to find information about eczema. Some parents found no information that seemed relevant to them while others, conversely, ‘got lost’ in a bewildering excess of information. There were also some confident users who felt able to appraise information and others who used the internet less for information and more for support.

We were surprised that not more of our interviewees had sought information from the internet, given that the literature suggests that the majority of patients do so.^12^ One possible explanation for this is that most of the interviewees were caring for children with mild eczema. Our finding that some people are becoming confused by excessive volumes of information while others are not finding any information of relevance, suggests that people do need help developing online health literacy or help in being directed towards high quality information online. Participants in this study said that they would welcome more signposting towards useful online resources by healthcare professionals. Other research has shown that it can be difficult to find specific information on the internet and that standardised labelling of sites could improve search engine results.^19^ It has also been suggested that directing patients or carers towards particular websites is one of the positive responses that professionals can make to concerns about potential difficulties posed by web-derived information presented by patients.^8^

Among this small sample we did not find any instances where the carers’ search for online information had challenged the doctor–patient relationship. Interviewees seemed to be searching for supplementary advice that they might not have received in a clinical encounter, echoing previous research findings suggesting that online information-seeking is generally viewed as additional to professional advice rather than replacing it.^8^ ^11^ ^20^

One strength of this study is that we used a population-based sampling frame (as almost all UK residents are registered with a GP) in order to seek a diverse range of views, yet we still have few single parents, fathers, ethnic minorities and low-income households, and a relatively high number of more educated parents. However, this makes our finding that people encountered substantial difficulties making sense of online information still more striking, as a more diverse group of participants could have reported still greater diversity in their experiences of information seeking.

A further limitation is that many of our interviewees had not sought information on the internet regarding eczema. The original aim of this study was to explore understandings of eczema diagnosis and treatment. We carried out an analysis of the data on information-seeking through the internet as this emerged as a recurring and interesting theme. However, further research focusing specifically on this topic is warranted.

## Conclusion

Even among carers of children with mild eczema, seeking information online commonly presented difficulties, with some findings of little relevance to them, while others felt bewildered by the volume of information available. Carers suggested that better signposting towards quality online information from health professionals would be helpful.

Healthcare professionals need to take every opportunity to direct patients and carers towards high quality information available on the internet. Health professionals should also remain aware during consultations that carers may already have sought information on the internet and be prepared to engage with this positively. Indeed, it may help to enquire about this in order to meet concerns and direct them towards quality online information. Although there are good sources of information about eczema available on the internet (box 2), remaining abreast of the wide range of online resources available can be a daunting task, particularly for generalist health practitioners. Well-developed clinical systems could make it easier for health professionals to identify high-quality evidence-based sites, for instance, by offering links to accredited information resources when diagnostic codes are entered.

Box 2 **Sources of information about eczema**

NHS Choices http://www.nhs.uk/conditions/Eczema-(atopic)

The National Eczema Society http://www.eczema.org

Nottingham Support Group for Carers of Children with Eczema http://www.nottinghameczema.org.uk
